# Neighborhood Environment and Social Support Received: An Examination of Race and Gender in Baltimore City

**DOI:** 10.1093/geroni/igab046.1838

**Published:** 2021-12-17

**Authors:** Sol Baik, Christine Mair, Amanda Lehning, Ji Hyang Cheon, Shari Waldstein, Michele Evans, Alan Zonderman

**Affiliations:** 1 University of Maryland, Baltimore, Baltimore, Maryland, United States; 2 University of Maryland, Baltimore County (UMBC), Baltimore, Maryland, United States; 3 University of Maryland, Baltimore County, University of Maryland Baltimore County, Maryland, United States; 4 University of Maryland, Baltimore County, Baltimore, Maryland, United States; 5 NIA, Baltimore, Maryland, United States; 6 HANDLS, Baltimore, Maryland, United States

## Abstract

Social support in urban settings is likely shaped by the context of the neighborhood environment. Patterns of support may also differ by the type of support received as well as characteristics of the person receiving support. For example, women and Black residents may have stronger support networks compared to men and white individuals, and variation by gender and race in social support may have important implications for promoting well-being in disadvantaged neighborhoods. To investigate the presence of these potential patterns in a disadvantaged urban environment, we analyzed 2,553 Baltimore City residents (ages 30-64) from the baseline wave (2004-2009) of the Healthy Aging in Neighborhoods of Diversity across the Life Span (HANDLS) study. We tested associations between self-assessed neighborhood environment (disorder, cohesion, and control) and social support (from partners, children, and/or friends) and further explored variation by intersections of race and gender using multi-group structural equation modeling. Our results suggest that individuals are more likely to receive support when they perceive their neighborhood to have higher social control and cohesion, particularly in terms of support from friends. Although interactions by race and sex were not statistically significant, a descriptive pattern emerged wherein Black women are particularly likely to receive support from multiple sources when they report more social control in their neighborhood. On the other hand, there is almost no association between neighborhood environment and social support for Black men. We discuss these findings in light of potential neighborhood inequities in informal support access in Baltimore City and similar urban settings.

